# Standalone deep learning versus experts for diagnosis lung cancer on chest computed tomography: a systematic review

**DOI:** 10.1007/s00330-024-10804-6

**Published:** 2024-05-22

**Authors:** Ting-Wei Wang, Jia-Sheng Hong, Hwa-Yen Chiu, Heng-Sheng Chao, Yuh-Min Chen, Yu-Te Wu

**Affiliations:** 1 Institute of Biophotonics, National Yang-Ming Chiao Tung University, Taipei, Taiwan; 2 School of Medicine, National Yang-Ming Chiao Tung University, Taipei, Taiwan; 3 Department of Chest Medicine, Taipei Veteran General Hospital, Taipei, Taiwan

**Keywords:** Deep learning, Lung neoplasms, Computed tomography (CT), Meta-analysis, Comparative study

## Abstract

**Purpose:**

To compare the diagnostic performance of standalone deep learning (DL) algorithms and human experts in lung cancer detection on chest computed tomography (CT) scans.

**Materials and methods:**

This study searched for studies on PubMed, Embase, and Web of Science from their inception until November 2023. We focused on adult lung cancer patients and compared the efficacy of DL algorithms and expert radiologists in disease diagnosis on CT scans. Quality assessment was performed using QUADAS-2, QUADAS-C, and CLAIM. Bivariate random-effects and subgroup analyses were performed for tasks (malignancy classification vs invasiveness classification), imaging modalities (CT vs low-dose CT [LDCT] vs high-resolution CT), study region, software used, and publication year.

**Results:**

We included 20 studies on various aspects of lung cancer diagnosis on CT scans. Quantitatively, DL algorithms exhibited superior sensitivity (82%) and specificity (75%) compared to human experts (sensitivity 81%, specificity 69%). However, the difference in specificity was statistically significant, whereas the difference in sensitivity was not statistically significant. The DL algorithms’ performance varied across different imaging modalities and tasks, demonstrating the need for tailored optimization of DL algorithms. Notably, DL algorithms matched experts in sensitivity on standard CT, surpassing them in specificity, but showed higher sensitivity with lower specificity on LDCT scans.

**Conclusion:**

DL algorithms demonstrated improved accuracy over human readers in malignancy and invasiveness classification on CT scans. However, their performance varies by imaging modality, underlining the importance of continued research to fully assess DL algorithms’ diagnostic effectiveness in lung cancer.

**Clinical relevance statement:**

DL algorithms have the potential to refine lung cancer diagnosis on CT, matching human sensitivity and surpassing in specificity. These findings call for further DL optimization across imaging modalities, aiming to advance clinical diagnostics and patient outcomes.

**Key Points:**

- *Lung cancer diagnosis by CT is challenging and can be improved with AI integration*.
- *DL shows higher accuracy in lung cancer detection on CT than human experts*.
- *Enhanced DL accuracy could lead to improved lung cancer diagnosis and outcomes*.

## Introduction

Lung cancer remains the leading cause of cancer death worldwide, with an estimated 2.38 million new cases and 1.27 million fatalities in 2023 [1]. Early detection through imaging, particularly through chest computed tomography (CT) scans, is crucial to improving patient outcomes [2]. However, interpreting CT scans can be challenging and subjective, depending on the radiologist’s experience and expertise [3]. To address these limitations in interpreting CT scan results, artificial intelligence (AI) models, particularly deep learning (DL) models, have been employed to enhance the accuracy of lung cancer diagnoses. DL models, especially convolutional neural networks, have been trained to identify subtle patterns in imaging data, with their accuracy potentially surpassing that of human experts [4, 5]. DL models offer a consistent and rapid analysis, which is particularly beneficial in managing large volumes of imaging data [6].

Applying AI in clinical practice poses challenges. Radiologists concerned about the “black box” nature of AI models question their transparency and analytical ability in clinical decision-making [7]. Moreover, the performance of DL models varies widely based on the quality of training data, signifying that DL models may be unreliable or biased [8]. Nonetheless, the integration of AI into radiological practice promises substantial benefits. AI can help reduce diagnostic errors, complement the skills of radiologists, and promote superior patient care [9]. AI can also alleviate the workload of radiologists, freeing their time for complex cases and patient interaction [10].

Previous meta-analyses have focused on the performance of AI alone in analyzing images captured through various imaging modalities [11–14]. Comparing the performance of AI models with that of radiologists can provide valuable insights. Accordingly, the aim of the present study was to conduct a meta-analysis to provide a comparative evaluation of the accuracy of expert radiologists and DL models in diagnosing lung cancer on chest CT scans. The study also reviewed and analyzed the literature to understand the strengths, limitations, and applications of DL models in lung cancer screening and diagnosis.

## Materials and methods

### General guidelines

This systematic review and meta-analysis rigorously adhered to the 2020 preferred reporting items for systematic reviews and meta-analyses (PRISMA) guidelines [15]. Fidelity to the PRISMA checklist was upheld during the study’s design and reporting stages to ensure methodological rigor. The extent of conformity to these guidelines is elaborated in Tables S1 and S2. Additionally, the study was officially registered in PROSPERO (registration number: CRD42023479874). The study did not involve direct clinical interactions with patients, obviating the need for ethical approval or informed consent.

### Database searches and identification of eligible manuscripts

Two analysts (T.W.W. and J.S.H.) searched the literature for studies that compared the effectiveness of DL models with that of expert assessment in diagnosing lung cancer on chest CT scans. They comprehensively searched three databases—PubMed, Embase, and Web of Science—from their inception until November 7, 2023. Table S3 provides a summary of our findings. We examined the titles and abstracts of the identified articles and included articles relevant to DL in evaluating CT scans. Any disagreements between the analysts were resolved by consulting a third specialist. Studies that focused on peri fissure nodule classification tasks focused on detection or segmentation tasks, did not involve CT, did not employ DL models, were not published in English, or did not provide contingency tables were excluded to ensure a concentrated, high-quality review; moreover, review articles were excluded.

### Data extraction and management

Data extraction was performed by two analysts (T.W.W. and J.S.H.). The extracted items were systematically categorized as follows: study country, study type, study design, reference standard for nodule identification, classification involved (malignancy classification or invasiveness classification), population (defined as the percentage of patients with a positive outcome), imaging modality, number of readers, and DL algorithms utilized. Outcome measures included the area under the receiver operating characteristic curve (AUC), sensitivity, and specificity. These measures were used to assess the performance of the human experts and DL algorithms; they were also used to assess the performance of the DL algorithms when applied alone. In the event that the performance measures for the DL algorithms and human experts were not explicitly provided in the included studies, we derived them from the available information if feasible.

### Quality assessment

The quality of the included studies was evaluated using the quality assessment of diagnostic accuracy studies 2 (QUADAS-2) framework [16] and the QUADAS Comparative (QUADAS-C) tool [17]. Additionally, the reporting quality of each study was assessed on the basis of the checklist for AI in medical imaging (CLAIM) [18]. This assessment was undertaken by the same analysts responsible for data extraction. Because of the complexity of assessing AI applications in medical imaging, any differences in interpretation were resolved by consulting with senior researchers involved in the study.

### Statistical analysis

In this study, 2 × 2 contingency tables were constructed for each included study by calculating true positives, false positives, true negatives, and false negatives on the basis of the data reported in the studies and their provided sensitivity and specificity values. We combined these data by computing pooled estimates and 95% confidence intervals (CIs) for the performance measures derived for radiologists and DL algorithms by employing bivariate random-effects models. In our analysis, both sensitivity and specificity values were transformed prior to inclusion in the model. Recognizing the bounded nature of these measures (ranging from 0 to 1), we applied a logit transformation to both sensitivity and specificity. Furthermore, summary receiver operating characteristic (SROC) curves were employed to compare the accuracy of the DL algorithms and human experts in the diagnostic process. Forest plots were generated to represent the results of the included studies visually according to classification task and image type subgroup. These plots depicted the studies’ collective results and segmented the results based on specific imaging modalities and tasks. Subgroup analysis was conducted to determine the effects of the study region, software used, and publication year on the performance of DL algorithms and human experts in malignancy classification on CT scans. Finally, to evaluate publication bias, a separate analysis for human experts and DL algorithms was conducted using Deeks’ funnel plot asymmetry test [19]. This statistical approach is designed to detect bias in meta-analyses; asymmetry in the funnel plot indicates publication bias in the published findings. Stata (version 18.0) with metadata [20] and Midas were used for statistical analysis.

## Results

### Study identification and selection

**Fig. 1:**
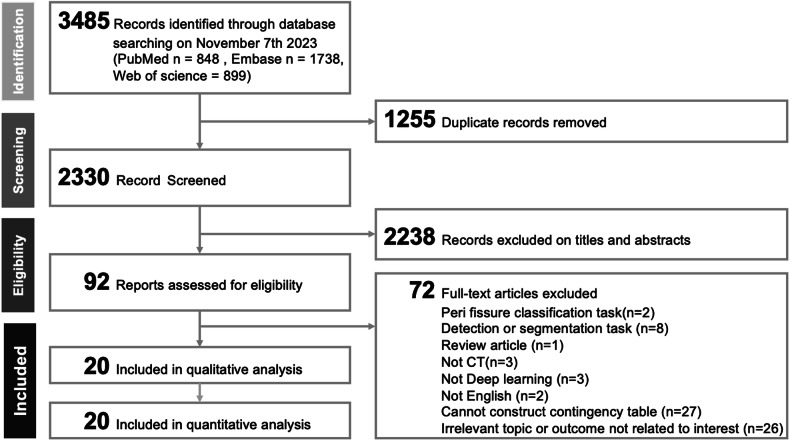
PRISMA flowchart of the search process for study inclusion

Figure 1 illustrates the PRISMA flowchart of the search process. The search yielded 3485 studies. After removing 1255 duplicates, we screened 2330 articles using EndNote, excluding 2238 on the basis of insufficient relevance. Further scrutiny of 92 full-text articles led to additional exclusions, as detailed in Table S4. Finally, we selected 20 eligible studies [21–40] for inclusion in our meta-analysis.

### Basic characteristics of included studies

Tables 1 and 2 provide a summary of the characteristics of the included studies. All but one study [39] utilized retrospective datasets. Additionally, 12 studies (11 using CT scans and one using low-dose CT [LDCT]) focused on malignancy classification. Seven studies focused on the classification of invasiveness (four using CT and three using high-resolution CT [HRCT]), and one study incorporated classifying both malignancies and invasiveness through the use of CT scans. Most of the studies were cohort studies, except one case-control study [21] and one study [26] that retrospectively analyzed data from a randomized controlled trial. All studies employed histopathology as the reference standard for diagnosis. The studies considered various nodule types, including subcentimeter nodules, ground-glass nodules, subsolid nodules, nonsolid nodules, small nodules, and nodules or adenomas.

**Table 1:** Basic characteristics of included studies

Author	Country	Study type	Study design	Reference standard	Nodule type
Liu et al [21]	China	Case-control	Retrospective	Surgical pathology or follow-up	Subcentimeter solid pulmonary nodules
Wang et al [22]	China	Cohort	Retrospective	Pathological diagnosis	Ground-glass nodules
Lv et al [23]	China	Cohort	Retrospective	Confirmed by the final pathology	Lung adenocarcinoma
Zhang et al [24]	China	Cohort	Retrospective	Pathology diagnoses	Non-solid nodules
Yanagawa [25]	Japan	Cohort	Retrospective	Pathology diagnoses	Non-mucinous adenocarcinoma
Venkadesh et al [26]	Netherlands	Randomized controlled trial	Retrospective	Histologic analysis–proven malignancies	Nodules
Sun et al [27]	China	Cohort	Retrospective	Histologically confirmed diagnosis	Nodules or mass
Park et al [28]	South Korea	Cohort	Retrospective	Pathological report	Subsolid nodules
Lv et al [29]	China	Cohort	Retrospective	Biopsy	Nodules
Coruh et al [30]	Turkey	Cohort	Retrospective	Proven histopathologically	Nodules
Gong et al [31]	China	Cohort	Retrospective	Histopathologically confirmed	Ground glass nodules
Yang et al [32]	China	Cohort	Retrospective	Biopsy or surgery proven	Nodules
Wang et al [33]	China	Cohort	Retrospective	Pathology assay	Pulmonary subsolid nodules
Wan et al [34]	Taiwan	Cohort	Retrospective	Pathologically proven	Nodules
Liu et al [35]	China	Cohort	Retrospective	Pathologically confirmed	Nodules
Kim et al [36]	South Korea	Cohort	Retrospective	Pathological diagnosis	Subsolid nodules
He et al [37]	China	Cohort	Retrospective	Pathological diagnosis	Nodules
Chae et al [38]	South Korea	Cohort	Retrospective	Pathologically proven	Small nodules (< 2 cm)
Zhang et al [39]	China	Cohort	Prospective	Pathologically confirmed	Nodules
Wang et al [40]	China	Cohort	Retrospective	Histologically confirmed	Ground glass nodules < 3 mm

**Table 2:** Comparative overview of AI algorithms in diagnostic studies

Author	Task	Population (% of positive)	Modality	No. of readers	AI algorithm
Liu et al [21]	Malignancy classification	200 (50%)	CT	2	Deepwise AI Lab
Wang et al [22]	Malignancy classification	63 (84.1%)	CT	2	DenseNet (in-house)
Lv et al [23]	Invasiveness classification	114 (53.5%)	HRCT	2	CNN (in-house)
Zhang et al [24]	Invasiveness classification	177 (33.9%)	HRCT	3	SE-ResNet (in-house)
Yanagawa [25]	Invasiveness classification	90 (51.1%)	CT	3	3D-CNN (in-house)
Venkadesh et al [26]	Malignancy classification	175 (33.7%)	CT	11	DL algorithm (open source)
Sun et al [27]	Malignancy classification	47 (59.6%)	CT	3	CNN (in-house)
Park et al [28]	Invasiveness classification	90 (50%)	CT	6	CNN (in-house)
Lv et al [29]	Malignancy classification	341 (76.5%)	CT	126	HONORS (in-house)
Coruh et al [30]	Malignancy classification	158 (48.7%)	CT	2	HuiYing Medical Technology Co., Ltd.
Gong et al [31]	Malignancy classification/invasiveness classification	202 (60.9%)/123 (30.0%)	CT	6	Two-stage DNN (in-house)
Yang et al [32]	Malignancy classification	220 (63.6%)	CT	4	Partial U-Net + CapNets (in-house)
Wang et al [33]	Invasiveness classification	1557 (44.4%)	CT	3	CNN (in-house)
Wan et al [34]	Malignancy classification	75 (62.7%)	LDCT	2	VS-CAD AI system
Liu et al (2020) [35]	Malignancy classification	247 (77.7%)	CT	2	σ-Discover/lung; 12 Sigma Technologies
Kim et al [36]	Invasiveness classification	101 (83.2%)	CT	3	2.5D DenseNet (in-house)
He et al [37]	Malignancy classification	50 (34%)	CT	3	CNN (in-house)
Chae et al [38]	Malignancy classification	60 (50%)	CT	6	CT-lungNET (in-house)
Zhang et al [39]	Malignancy classification	50 (25%)	CT	25	CNN (in-house)
Wang et al [40]	Invasiveness classification	200 (44%)	HRCT	3	CNN (in-house)

*CT* computed tomography, *LDCT* low-dose computed tomography, *HRCT* high-resolution computed tomography

Several DL algorithms were employed in the 20 studies we reviewed. Specifically, five studies (25%) utilized commercially available AI algorithms, one employed open-source software, and the remaining 14 used customized algorithms developed in-house. This variation in DL models reflects the breadth of AI-based approaches available to improve diagnostic accuracy in medical imaging.

### Quality assessment

Figure S1 meticulously delineates the quality assessments of the incorporated studies, employing the QUADAS-2 and QUADAS-C instruments. Concurrently, Supplementary Table S5 offers an exhaustive elucidation of the potential biases and applicability issues pertinent to these studies.

In a rigorous assessment presented in Supplementary Table S6, twenty studies were evaluated against the CLAIM criteria. The mean CLAIM score attained by these studies was 30.45, corresponding to approximately 72.5% of the maximal attainable score of 42. This evaluation noted a standard deviation of 4.07, with individual study scores ranging from 24.00 to 36.00. A detailed dissection of the average scores obtained in various subsections of the CLAIM criteria for these studies is as follows: the title/abstract section attained a perfect score of 2 out of 2 (100%), the introduction section scored 1.95 out of 2 (97.5%), the methods section achieved 20.5 out of 28 (71.6%), the results section garnered 3.4 out of 5 (68%), the discussion section scored 1.85 out of 2 (92.5%), and the section encompassing other information attained 1.2 out of 3 (40%). This segmentation of scores elucidates the differential levels of comprehensiveness and quality across various segments of the evaluated studies.

### Outcome measures for standalone DL algorithms and human experts

In our meta-analysis, we derived pooled estimates for the sensitivity and specificity of human experts and DL algorithms in diagnosing lung cancer on chest CT scans (Table 3). The DL algorithms exhibited slightly higher sensitivity levels (82%; 95% CI: 79–86) than did the human experts (81%, 95% CI: 78–85), although the difference (2%) was nonsignificant (*p* = 0.06). Notably, the DL algorithms exhibited significantly higher specificity levels (75%, 95% CI: 70–80) than did the human experts (68%, 95% CI: 62–74), and the difference (7%) was significant (*p* < 0.01). These pooled estimates and the corresponding forest plots are presented in Fig. S2, and the SROC plot is displayed in Fig. S3. We noted a minor publication bias in the results for the DL algorithms (*p* = 0.01, Fig. S4a) but not in those for the human experts (Fig. S4b).

**Table 3:** Pooled estimates of performance measures for human experts and standalone DL algorithms for all included studies and tasks involving various imaging modalities

	Sensitivity				Specificity			
	Radiologist	*p* value	DL	*p* value	Radiologist	*p* value	DL	*p* value
All studies	0.81 (0.76–0.84)	< 0.01	0.82 (0.79–0.86)	< 0.01	0.69 (0.62–0.74)	< 0.01	0.75 (0.70–0.80)	< 0.01
Malignancy classification (*n* = 13)	0.81 (0.76–0.86)	< 0.01	0.83 (0.78–0.87)	< 0.01	0.61 (0.52–0.70)	< 0.01	0.73 (0.65–0.80)	0.02
CT (*n* = 12)	0.81 (0.75–0.85)	< 0.01	0.82 (0.77–0.86)	< 0.01	0.60 (0.50–0.70)	< 0.01	0.75 (0.66–0.82)	0.04
LDCT (*n* = 1)	0.89 (0.77–0.96)	< 0.01	0.94 (0.82–0.98)	< 0.01	0.82 (0.64–0.92)	< 0.01	0.39 (0.23–0.58)	0.26
Invasiveness classification (*n* = 8)	0.78 (0.73–0.83)	< 0.01	0.81 (0.76–0.85)	< 0.01	0.77 (0.70–0.82)	< 0.01	0.80 (0.74–0.85)	< 0.01
CT (*n* = 5)	0.76 (0.69–0.82)	< 0.01	0.79 (0.72–0.84)	< 0.01	0.78 (0.69–0.84)	< 0.01	0.81 (0.74–0.87)	< 0.01
HRCT (*n* = 3)	0.82 (0.74–0.88)	< 0.01	0.84 (0.77–0.90)	< 0.01	0.79 (0.67–0.68)	< 0.01	0.77 (0.64–0.86)	< 0.01

*CT* computed tomography, *LDCT* low-dose computed tomography, *HRCT* high-resolution computed tomography

**Fig. 2:**
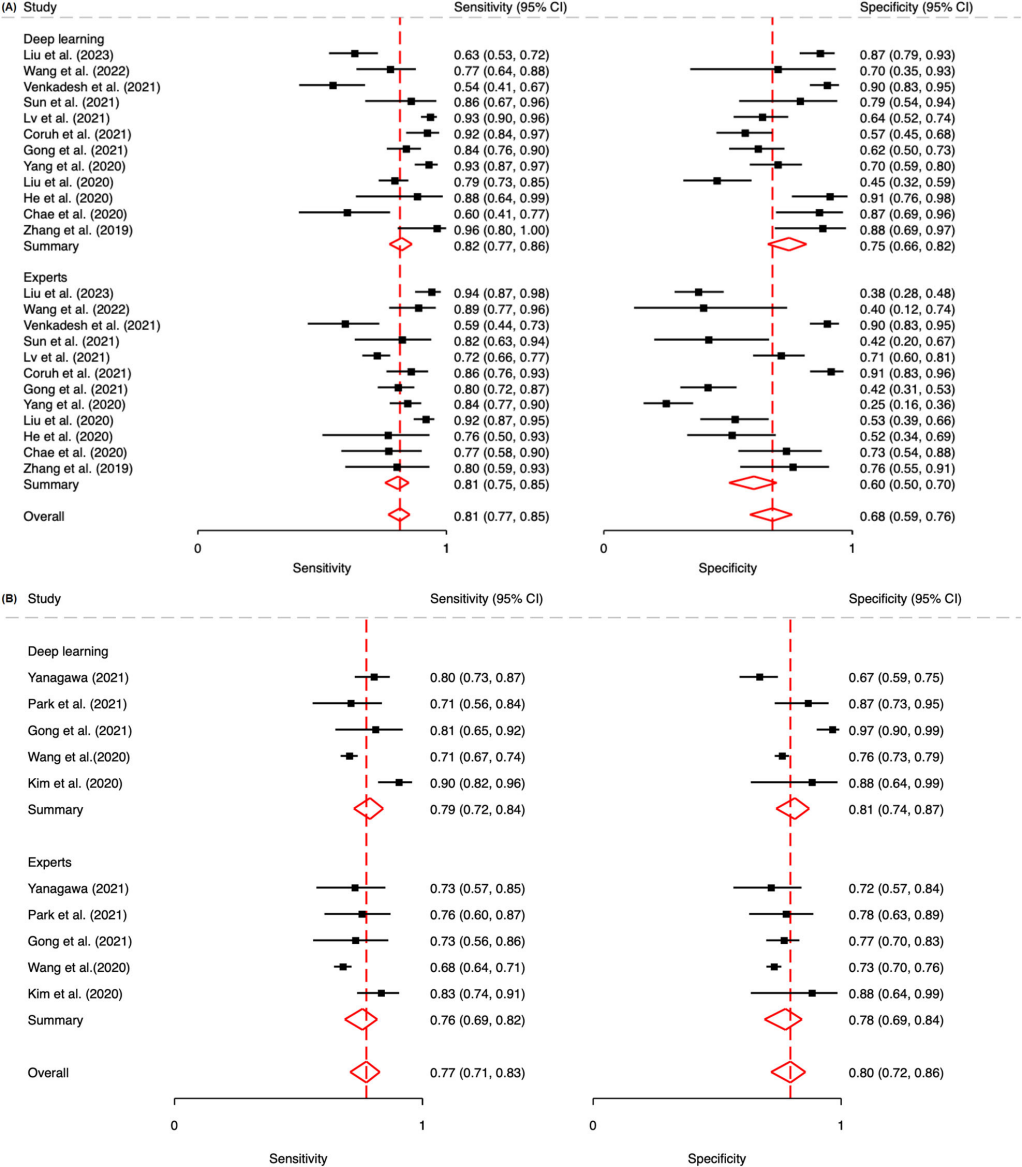
Forest plots showing pooled estimates for (**A**) malignancy classification on CT scans and (**B**) invasiveness classification on CT scans

**Fig. 3:**
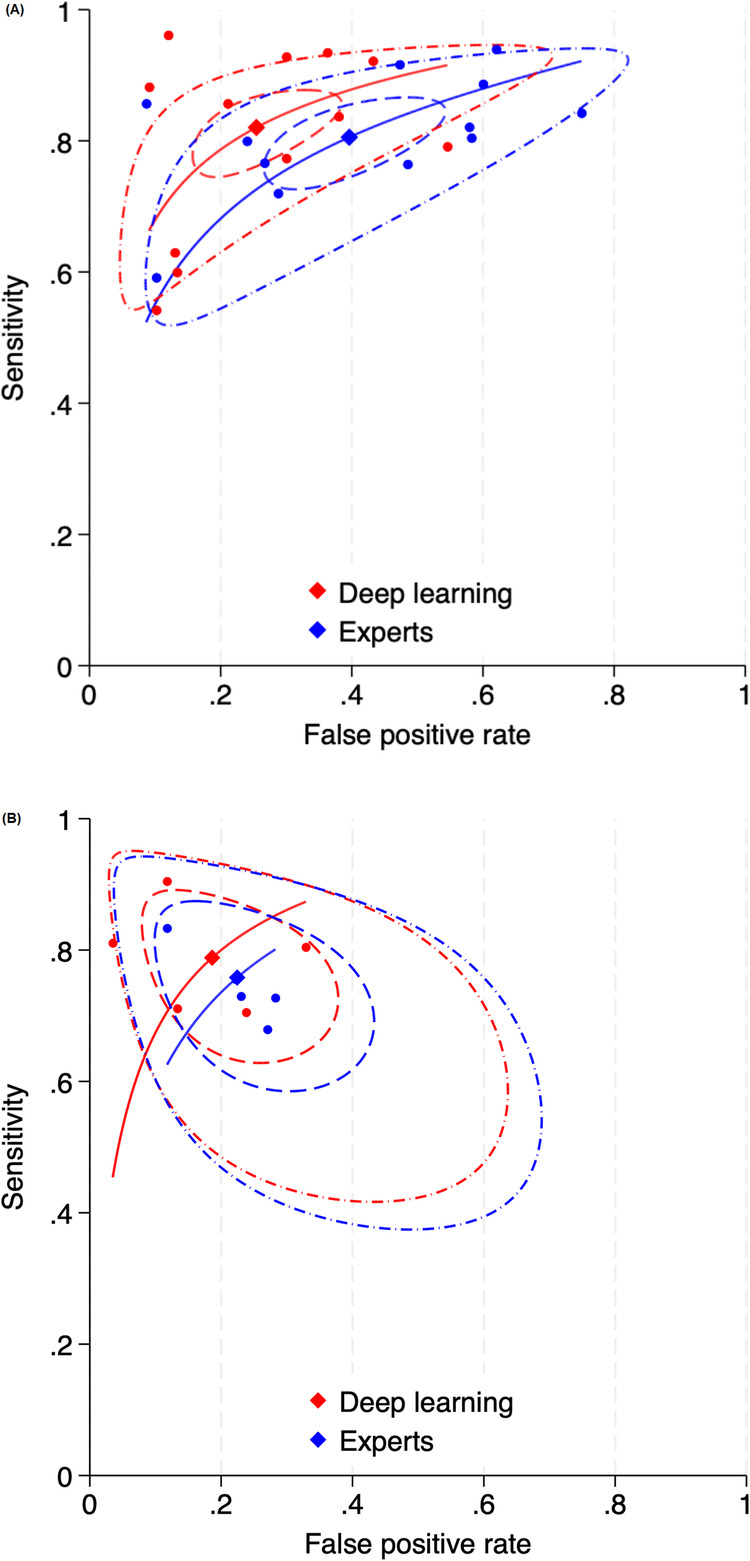
SROC curves indicating the performance of human experts (blue) and DL algorithms (red) in (**A**) malignancy classification on CT scans and (**B**) invasiveness classification on CT scans

**Fig. 4:**
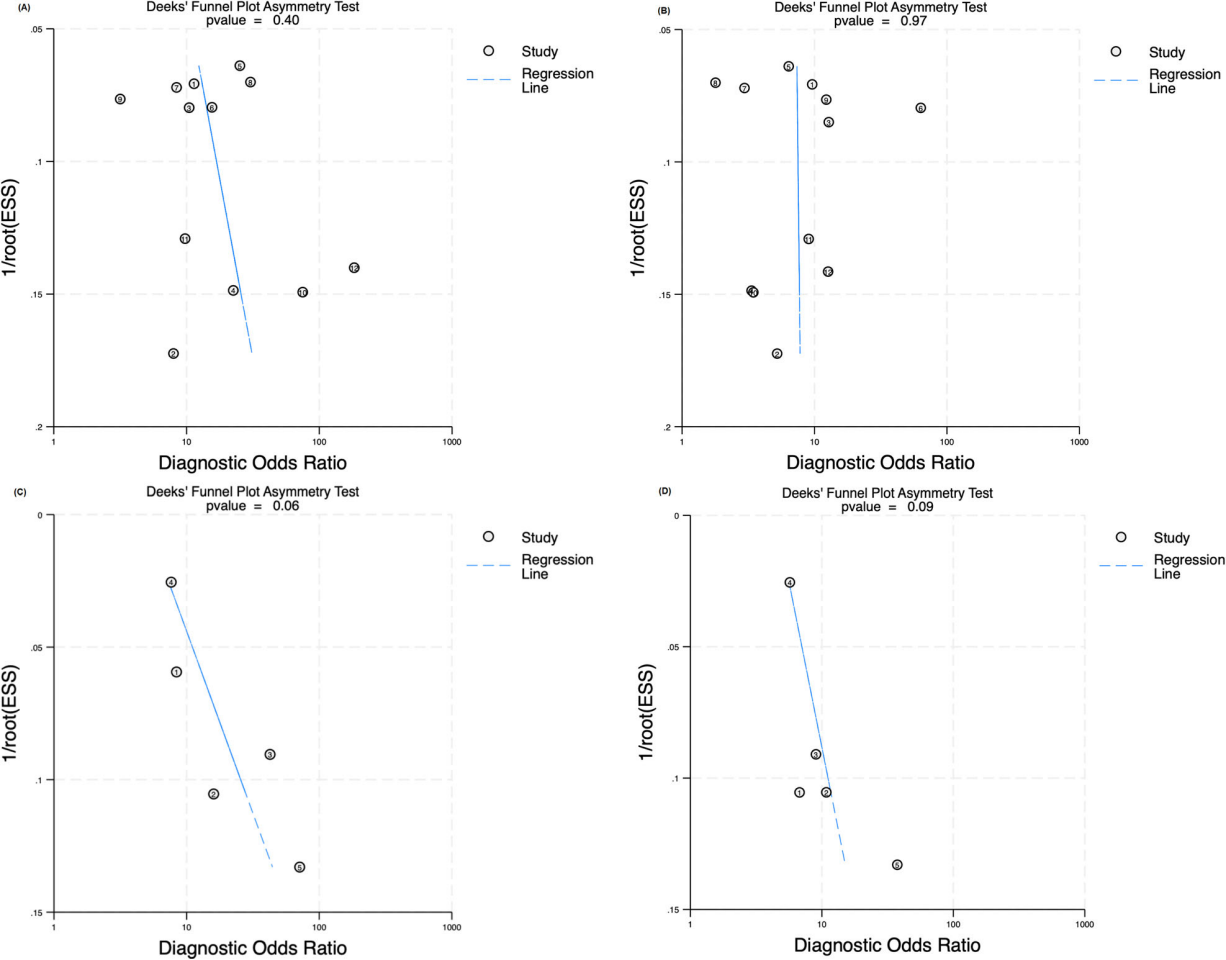
Deeks’ test plot shows (**A**) expert malignancy classification on CT scans, (**B**) DL algorithm malignancy classification on CT scans, (**C**) expert invasiveness classification on CT scans, and (**D**) DL algorithm invasiveness classification on CT scans. ESS, effective sample size

For the malignancy classification task, we observed notable differences between the DL algorithms and human experts in terms of their performance in diagnosing lung cancer on CT scans (Figs. 2a and 3a). Specifically, in the 12 studies on malignancy classification using CT, the DL algorithms and human experts demonstrated comparable sensitivity levels, which were 82% (95% CI: 77–86) and 81% (95% CI: 75–85), respectively. However, the specificity levels of the DL algorithms and human experts differed significantly, which were 75% (95% CI: 66–82) and 60% (95% CI: 50–70), respectively. No significant publication bias was noted in the results for the human experts or DL algorithms (Fig. 4a, b) in these studies. In the studies using LDCT, the sensitivity of the DL algorithms was superior to that of the human experts (94% [95% CI: 82–98] vs 89% [95% CI: 77–96]), but their specificity was inferior to that of the human experts (39% [95% CI: 23–58] vs 82% [95% CI: 64–92]).

For the invasiveness classification task, the DL algorithms demonstrated marginally higher sensitivity and specificity levels than did the human experts using CT (Figs. 2b and 3b). In the HRCT-based studies, the sensitivity and specificity of the DL algorithms and human experts were closely matched, showing no significant differences. These findings, as illustrated in the forest plots and tables, demonstrate the effectiveness of DL algorithms relative to traditional radiological analysis in lung cancer diagnosis.

This study also investigated potential confounders in malignancy classification on CT scans. Our analysis revealed significant regional differences in the performance of the human experts. However, we found no significant impact of study region, software, or publication year on the sensitivity and specificity of the DL algorithms. These results are presented in Table 4.

**Table 4:** Comparison of sensitivity and specificity of human experts and standalone DL algorithms for various moderators

Moderator	Category	*N*	DL				Radiologist			
			Sensitivity	*p* value	Specificity	*p* value	Sensitivity	*p* value	Specificity	*p* value
Region	Asian	10	0.84 (0.75–0.90)	0.65	0.76 (0.65–0.84)	0.89	0.84 (0.78–0.89)	0.22	0.51 (0.41–0.61)	< 0.01
	Europe	2	0.78 (0.52–0.92)		0.77 (0.54–0.91)		0.75 (0.57–0.87)		0.91 (0.79–0.96)	
Software	Commercially available	3	0.80 (0.65–0.69)	0.11	0.66 (0.48–0.80)	0.18	0.91 (0.86–0.84)	< 0.01	0.60 (0.45–0.93)	0.11
	Open source	1	0.54 (0.41–0.67)		0.90 (0.83–0.95)		0.59 (0.44–0.73)		0.90 (0.83–0.95)	
	In house	8	0.86 (0.79–0.91)		0.77 (0.67–0.85)		0.79 (0.74–0.83)		0.52 (0.36–0.67)	
Publication year	After 2021	7	0.82 (0.70–0.89)	0.71	0.75 (0.62–0.85)	0.77	0.82 (0.74–0.88)	0.73	0.64 (0.45–0.80)	0.57
	Before 2021	5	0.84 (0.70–0.93)		0.78 (0.62–0.68)		0.84 (0.74–0.90)		0.56 (0.33–0.76)	

## Discussion

This comprehensive meta-analysis compared the performance of standalone DL algorithms with that of human experts in lung cancer diagnosis on chest CT, including LDCT and HRCT scans. The study findings provide insights into the effectiveness of the integration of AI in medical imaging.

Our assessment of the diagnostic performance of DL algorithms versus human experts in lung cancer detection on chest CT scans revealed major differences in sensitivity and specificity. The sensitivity of the DL algorithms (82%; 95% CI: 79–86) was marginally higher than that (80%; 95% CI: 76–84) of the human experts, although this 2% difference was not statistically significant (*p* = 0.06). However, the DL algorithms achieved a significantly higher specificity level (75%; 95% CI: 70–80) than did the human experts (69%; 95% CI: 62–74), a difference of 6% (*p* < 0.01). This difference may be due to the DL algorithms’ ability to reduce false-positive diagnoses, significantly improving their efficiency in lung cancer screening. Moreover, our analysis revealed that the DL algorithms’ performance varied depending on the imaging modality and task. The DL algorithms demonstrated comparable sensitivity and higher specificity than did the human experts regarding malignancy classification on standard CT scans. However, on LDCT scans, the DL algorithms exhibited higher sensitivity but significantly lower specificity than the human experts. This variability in performance across different modalities and tasks highlights the necessity of tailoring the optimization of DL algorithms to ensure their efficacy and reliability in varied diagnostic scenarios.

Integrating AI into clinical diagnostics presents challenges and opportunities. A primary challenge is AI’s “black box” nature. Because the decision-making mechanisms employed by AI are not transparent, concerns arise regarding the interpretability and trustworthiness of AI-derived conclusions. This lack of transparency is critical in sensitive fields such as oncology, where diagnostic accuracy is crucial. Another challenge lies in the dependency of DL models on diverse and high-quality training data. Specifically, DL models require diverse, high-quality datasets for training and validation to enhance their reliability and reduce biases. This training can only be performed by humans who understand the intricacies of the data. Additionally, our study revealed experts’ performance inconsistencies across regions, highlighting an area where AI may be of assistance to human judgment. These results suggest that in the process of diagnosing lung cancer on CT scans, humans may benefit AI as much as AI benefits humans.

DL models can considerably supplement the expertise of radiologists. By managing routine tasks and streamlining analysis, AI can reduce radiologists’ workload, enabling them to devote more attention to complex cases and patient interaction. Moreover, AI’s ability to make predictions from individual cancer nodules may enable personalized treatments, including recommendations for surgery or observation, thus enhancing patient care.

This study has several limitations. First, because this study exclusively included noninterventional studies, AI assessments were never directly involved in diagnosing patients, raising the question of whether AI can detect cancer reliably in clinical practice. Second, our analysis was constrained to aggregated data; this is because individual patient data from the included studies were inaccessible, limiting our ability to perform more detailed, patient-specific evaluations. Third, we could not evaluate the relative levels of expertise of the radiologists whose work we reviewed. Finally, the predominance of retrospective studies in our analysis, as opposed to prospective, multicenter studies, may affect the generalizability and applicability of our findings to future clinical practice. These limitations highlight areas for further research, emphasizing the need for more integrative, patient-specific, and prospective studies to validate the efficacy of AI in clinical diagnostic settings.

## Conclusions

The results of our meta-analysis reveal that DL algorithms can accurately diagnose lung cancer on CT scans. The evaluated DL algorithms achieved similar sensitivity levels to human experts and greater specificity levels. However, further research is necessary to determine the effectiveness of DL models in classifying malignancies on LDCT scans and classifying invasiveness on HRCT scans. As AI continues to develop, its integration into oncology diagnostics can significantly enhance accuracy and patient outcomes, provided that it is balanced with human expertise.

## Supplementary information

ELECTRONIC SUPPLEMENTARY MATERIAL

## Abbreviations

AI: Artificial intelligence
AUC: Area under the receiver operating characteristic curve
CI: Confidence intervals
CLAIM: Checklist for artificial intelligence in medical imaging
CT: Computed tomography
DL: Deep learning
HRCT: High-resolution computed tomography
LDCT: Low-dose computed tomography
PRISMA: Preferred reporting items for systematic reviews and meta-analyses
QUADAS-2: Quality assessment of diagnostic accuracy studies 2
QUADAS-C: Quality assessment of diagnostic accuracy studies comparative
SROC: Summary receiver operating characteristic
