# Supplementary material for: Standalone deep learning versus experts for diagnosis lung cancer on chest computed tomography: a systematic review
Source: Eur Radiol. 2024 May 22;34(11):7397–407. doi: 10.1007/s00330-024-10804-6 (PMC11519296; doi:10.1007/s00330-024-10804-6)
Supplement: Supplementary file 1 — ELECTRONIC SUPPLEMENTARY MATERIAL [file 330_2024_10804_MOESM1_ESM.pdf]

Standalone Deep Learning versus Experts for Diagnosis Lung Cancer on Chest Computed Tomography: A Systematic Review

ELECTRONIC SUPPLEMENTARY MATERIAL

Table S1. PRISMA-DTA Abstract Checklist.

Section/Topic	Number	PRISMA-DTA for Abstracts Checklist Item	Reported on Page #
		TITLE and PURPOSE	
Title	1	Identify the report as a systematic review (+/- meta-analysis) of diagnostic test accuracy (DTA) studies.	Title page
Objectives	2	Indicate the research question, including components such as participants, index test, and target conditions.	1
		METHODS	
Eligibility criteria	3	Include study characteristics used as criteria for eligibility.	1
Information sources	4	List the key databases searched and the search dates.	1
Risk of bias & applicability	5	Indicate the methods of assessing risk of bias and applicability.	1
Synthesis of results	A1		1
		RESULTS	
Included studies	6	Indicate the number and type of included studies and the participants and relevant characteristics of the studies (including the reference standard).	1
Synthesis of results	7	Include the results for the analysis of diagnostic accuracy, preferably indicating the number of studies and participants. Describe test accuracy including variability; if meta-analysis was done, include summary results and confidence intervals.	1
		DISCUSSION	
Strengths and limitations	9	Provide a brief summary of the strengths and limitations of the evidence	1
Interpretation.	10	Provide a general interpretation of the results and the important implications.	1
		OTHER	
Funding	11	Indicate the primary source of funding for the review	NA
Registration	12	Provide the registration number and the registry name	NA

Adapted From: McInnes MDF, Moher D, et al. The PRISMA-DTA Group (2018). Preferred Reporting Items for a Systematic Review and Meta-analysis of Diagnostic Test Accuracy Studies: The PRISMA-DTA Statement. JAMA. 2018 Jan 23;319(4):388-396. doi: 10.1001/jama.2017.19163.

Table S2. PRISMA-DTA Checklist.

Section/Topic	Number	PRISMA-DTA for Abstracts Checklist Item	Reported on Page #
		TITLE and PURPOSE	
Title	1	Identify the report as a systematic review (+/- meta-analysis) of diagnostic test accuracy (DTA) studies.	Title page
Abstract	2	Abstract: See PRISMA-DTA for abstracts.	1
		INTRODUCTION	
Rationale	3	Describe the rationale for the review in the context of what is already known.	2
Clinical role of index test	D1	State the scientific and clinical background, including the intended use and clinical role of the index test, and if applicable, the rationale for minimally acceptable test accuracy (or minimum difference in accuracy for comparative design).	2
Objectives	4	Provide an explicit statement of question(s) being addressed in terms of participants, index test(s), and target condition(s).	2
		METHODS	
Protocol and registration	5	Indicate if a review protocol exists, if and where it can be accessed (e.g., Web address), and, if available, provide registration information including registration number.	3
Eligibility criteria	6	Specify study characteristics (participants, setting, index test(s), reference standard(s), target condition(s), and study design) and report characteristics (e.g., years considered, language, publication status) used as criteria for eligibility, giving rationale.	3
Information sources	7	Describe all information sources (e.g., databases with dates of coverage, contact with study authors to identify additional studies) in the search and date last searched.	3
Search	8	Present full search strategies for all electronic databases and other sources searched, including any limits used, such that they could be repeated.	3
Study selection	9	State the process for selecting studies (i.e., screening, eligibility, included in systematic review, and, if applicable, included in the meta-analysis).	3
Data collection process	10	Describe method of data extraction from reports (e.g., piloted forms, independently, in duplicate) and any processes for obtaining and confirming data from investigators.	3
Definitions for data extraction	11	Provide definitions used in data extraction and classifications of target condition(s), index test(s), reference standard(s) and other characteristics (e.g. study design, clinical setting).	3
Risk of bias and applicability	12	Describe methods used for assessing risk of bias in individual studies and concerns regarding the applicability to the review question.	4
Diagnostic accuracy measures	13	State the principal diagnostic accuracy measure(s) reported (e.g. sensitivity, specificity) and state the unit of assessment (e.g. per-patient, per-lesion).	4
Synthesis of results	14	Describe methods of handling data, combining results of studies and describing variability between studies. This could include, but is not limited to: a) handling of multiple definitions of target condition. b) handling of multiple thresholds of test positivity, c) handling multiple index test readers, d) handling of indeterminate test results, e) grouping and comparing tests, f) handling of different reference standards	4
Meta-analysis	D2	Report the statistical methods used for meta-analyses, if performed.	4
Additional analyses	16	Describe methods of additional analyses (e.g., sensitivity or subgroup analyses, meta-regression), if done, indicating which were pre-specified.	4
		RESULTS	
Study selection	17	Provide numbers of studies screened, assessed for eligibility, included in the review (and included in meta-analysis, if applicable) with reasons for exclusions at each stage, ideally with a flow diagram	5
Study characteristics	18	For each included study provide citations and present key characteristics including: a) participant characteristics (presentation, prior testing), b) clinical setting, c) study design, d) target condition definition, e) index test, f) reference standard, g) sample size, h) funding sources	5
Risk of bias and applicability	19	Present evaluation of risk of bias and concerns regarding applicability for each study.	5
Results of individual studies	20	For each analysis in each study (e.g. unique combination of index test, reference standard, and positivity threshold) report 2x2 data (TP, FP, FN, TN) with estimates of diagnostic accuracy and confidence intervals, ideally with a forest or receiver operator characteristic (ROC) plot.	NA
Synthesis of results	21	Describe test accuracy, including variability; if meta-analysis was done, include results and confidence intervals	6

Additional analysis	23	Give results of additional analyses, if done (e.g., sensitivity or subgroup analyses, meta-regression; analysis of index test: failure rates, proportion of inconclusive results, adverse events).	6
		DISCUSSION	
Summary of evidence	24	Summarize the main findings including the strength of evidence	7-8
Limitations	25	Discuss limitations from included studies (e.g. risk of bias and concerns regarding applicability) and from the review process (e.g. incomplete retrieval of identified research).	7-8
Conclusions	26	Provide a general interpretation of the results in the context of other evidence. Discuss implications for future research and clinical practice (e.g. the intended use and clinical role of the index test)	8
		OTHER	
Funding	27	For the systematic review, describe the sources of funding and other support and the role of the funders	8

Adapted From: McInnes MDF, Moher D, et al. The PRISMA-DTA Group (2018). Preferred Reporting Items for a Systematic Review and Meta-analysis of Diagnostic Test Accuracy Studies: The PRISMA-DTA Statement. JAMA. 2018 Jan 23;319(4):388-396. doi: 10.1001/jama.2017.19163.

Table S3. Keywords and search results in different database

Database	Keyword	Date	Results
PubMed	(lung neoplasm OR pulmonary carcinoma OR lung cancer OR lung carcinoma OR lung nodule) AND (segmentation OR contouring) AND (deep learning OR convolutional neural networks OR CNN) AND (computed tomography OR CT scan OR LDCT OR low dose CT OR low-dose computed tomography)	2023/11/07	848
Embase	(lung neoplasm OR pulmonary carcinoma OR lung cancer OR lung carcinoma OR lung nodule) AND (segmentation OR contouring) AND (deep learning OR convolutional neural networks OR CNN) AND (computed tomography OR CT scan OR LDCT OR low dose CT OR low-dose computed tomography)	2023/11/07	1738
Web of Science	(lung neoplasm OR pulmonary carcinoma OR lung cancer OR lung carcinoma OR lung nodule) AND (segmentation OR contouring) AND (deep learning OR convolutional neural networks OR CNN) AND (computed tomography OR CT scan OR LDCT OR low dose CT OR low-dose computed tomography)	2023/11/07	899

Table S4. Excluded article and reason

Title	Exclude
Deep learning-based computer-aided diagnostic models versus other methods for predicting malignancy risk in CT-detected pulmonary nodules	Review
Radiologists with and without deep learning–based computer-aided diagnosis: comparison of performance and interobserver agreement for characterizing and diagnosing pulmonary nodules/masses	Cannot construct contingency table
Deep Learning-Based Digitally Reconstructed Tomography of the Chest in the Evaluation of Solitary Pulmonary Nodules: A Feasibility Study	Not CT
Deep learning predicts malignancy and metastasis of solid pulmonary nodules from CT scans	Cannot construct contingency table
Sybil: A Validated Deep Learning Model to Predict Future Lung Cancer Risk From a Single Low-Dose Chest Computed Tomography	Cannot construct contingency table
P1.20-01 Computed Tomography-based Deep Learning Model for Spread Through Air Spaces Prediction in Ground-Glass Predominant Lung Adenocarcinoma	Outcome not relate to interest
Generative Adversarial Network-Based Image Conversion Among Different Computed Tomography Protocols and Vendors: Effects on Accuracy and Variability in Quantifying Regional Disease Patterns of Interstitial Lung Disease	Outcome not relate to interest
EP04.01-11 AI-based Detection on Low-Dose CT: A Focus on Augmenting Model Performance	Cannot construct contingency table
A Novel Artificial Intelligence Based Denoising Method for Ultra-Low Dose CT Used for Lung Cancer Screening	Outcome not relate to interest
MS-Net: Learning to assess the malignant status of a lung nodule by a radiologist and her peers	Outcome not relate to interest
Assessment of artificial intelligence-aided computed tomography in lung cancer screening	Cannot construct contingency table
ViSTA: A Novel Network Improving Lung Adenocarcinoma Invasiveness Prediction from Follow-Up CT Series	Outcome not relate to interest
Lung Nodule Detectability of Artificial Intelligence-assisted CT Image Reading in Lung Cancer Screening	Not deep learning
Performance of an Artificial Intelligence-Based Platform Against Clinical Radiology Reports for the Evaluation of Noncontrast Chest CT	Outcome not relate to interest
Analysis of the Causes of Solitary Pulmonary Nodule Misdiagnosed as Lung Cancer by Using Artificial Intelligence: A Retrospective Study at a Single Center	Outcome not relate to interest
Development and validation of an abnormality-derived deep-learning diagnostic system for major respiratory diseases	Cannot construct contingency table
Validation of a deep learning computer aided system for CT based lung nodule detection, classification, and growth rate estimation in a routine clinical population	Cannot construct contingency table
Automated Computer-Aided Detection of Lung Nodules in Metastatic Colorectal Cancer Patients for the Identification of Pulmonary Oligometastatic Disease	Detection task
Deep Learning-based Artificial Intelligence Improves Accuracy of Error-prone Lung Nodules	Outcome not relate to interest
Development of deep learning-assisted overscan decision algorithm in low-dose chest CT: Application to lung cancer screening in Korean National CT accreditation program	Outcome not relate to interest
Asbestosis diagnosis algorithm combining the lung segmentation method and deep learning model in computed tomography image	Outcome not relate to interest
Development and performance evaluation of a deep learning lung nodule detection system	Cannot construct contingency table

A radiomics-based decision support tool improves lung cancer diagnosis in combination with the Herder score in large lung nodules	Not deep learning
Comparison of two reader modes of computer-aided diagnosis in lung nodules on low-dose chest CT scan	Cannot construct contingency table
Higher agreement between readers with deep learning CAD software for reporting pulmonary nodules on CT	Not deep learning
Pulmonary nodules detection assistant platform: An effective computer aided system for early pulmonary nodules detection in physical examination	Cannot construct contingency table
Performance of a deep learning-based lung nodule detection system as an alternative reader in a Chinese lung cancer screening program	Cannot construct contingency table
Recognition of Peripheral Lung Cancer and Focal Pneumonia on Chest Computed Tomography Images Based on Convolutional Neural Network	Cannot construct contingency table
Clinical impact of a deep learning system for automated detection of missed pulmonary nodules on routine body computed tomography including the chest region	Detection task
Diagnostic Accuracy and Performance of Artificial Intelligence in Detecting Lung Nodules in Patients With Complex Lung Disease: A Noninferiority Study	Outcome not relate to interest
Comparison on convolutional neural network classification model and radiologists in differentiating invasive lung adenocarcinoma	Not English
Use of a Dual Artificial Intelligence Platform to Detect Unreported Lung Nodules	Outcome not relate to interest
Attribute prediction of concurrent nodules in lung CT in patients with lung adenocarcinoma using three-dimensional convolutional neural network	Segmentation task
Towards radiologist-level cancer risk assessment in CT lung screening using deep learning	Cannot construct contingency table
Estimation of malignancy of pulmonary nodules at CT scans: Effect of computer-aided diagnosis on diagnostic performance of radiologists	Cannot construct contingency table
Predictive value of a novel Asian lung cancer screening nomogram based on artificial intelligence and epidemiological characteristics	Outcome not relate to interest
Exploring heat maps for explaining an artificial intelligence tool for lung cancer detection	Cannot construct contingency table
Pulmonary Nodule Classification in Lung Cancer from 3D Thoracic CT Scans Using fastai and MONAI	Cannot construct contingency table
Deep learning for lung cancer detection on screening ct scans: Results of a large-scale public competition and an observer study with 11 radiologists	Cannot construct contingency table
Evaluation of a novel deep learning-based classifier for perifissural nodules	peri-fissure nodules classification
Clinical validation of a segmentation tool for pulmonary nodules in lung cancer screening	Outcome not relate to interest
Automated detection of lung nodules and coronary artery calcium using artificial intelligence on low-dose CT scans for lung cancer screening: accuracy and prognostic value	Detection task
Use of a Commercially Available Deep Learning Algorithm to Measure the Solid Portions of Lung Cancer Manifesting as Subsolid Lesions at CT: Comparisons with Radiologists and Invasive Component Size at Pathologic Examination	Outcome not relate to interest
A cross-modal 3D deep learning for accurate lymph node metastasis prediction in clinical stage T1 lung adenocarcinoma	Outcome not relate to interest

Deep Learning in CT Images: Automated Pulmonary Nodule Detection for Subsequent Management Using Convolutional Neural Network	Outcome not relate to interest
Artificial intelligence based on deep learning for differential diagnosis between benign and malignant pulmonary nodules: A real-world, multicenter, diagnostic study	Cannot construct contingency table
Comparison and Fusion of Deep Learning and Radiomics Features of Ground-Glass Nodules to Predict the Invasiveness Risk of Stage-I Lung Adenocarcinomas in CT Scan	Cannot construct contingency table
A robust convolutional neural network for lung nodule detection in the presence of foreign bodies	Not CT
Deep learning-based detection system for multiclass lesions on chest radiographs: comparison with observer readings	Not CT
Attribute-guided image generation of three-dimensional computed tomography images of lung nodules using a generative adversarial network	Outcome not relate to interest
Efficiency of a computer-aided diagnosis (CAD) system with deep learning in detection of pulmonary nodules on 1-mm-thick images of computed tomography	Detection task
Discrimination between transient and persistent subsolid pulmonary nodules on baseline CT using deep transfer learning	Outcome not relate to interest
DeepLN: An artificial intelligence-based automated system for lung cancer screening	Cannot construct contingency table
A deep residual learning network for predicting lung adenocarcinoma manifesting as ground-glass nodule on CT images	Cannot construct contingency table
Deep-learning-based model observer for a lung nodule detection task in computed tomography	Outcome not relate to interest
Validation of a deep learning-based computer-aided system for lung nodule detection in a Chinese lung cancer screening program	Cannot construct contingency table
Development and clinical application of deep learning model for lung nodules screening on CT images	Detection task
Dual-branch residual network for lung nodule segmentation	Outcome not relate to interest
An artificial intelligence pathological diagnosis model for lung cancer based on deep learning algorithm: Development and application	Not English
Automatic Lung Nodule Detection Combined With Gaze Information Improves Radiologists' Screening Performance	Outcome not relate to interest
IILS: Intelligent imaging layout system for automatic imaging report standardization and intra-interdisciplinary clinical workflow optimization	Cannot construct contingency table
Automated detection and segmentation of lung tumors using deep learning	Outcome not relate to interest
Evaluating a fully automated pulmonary nodule detection approach and its impact on radiologist performance	Cannot construct contingency table
[Performance of Deep-learning-based Artificial Intelligence on Detection of Pulmonary Nodules in Chest CT]	Cannot construct contingency table
Evaluating the performance of a deep learning-based computer-aided diagnosis (DL-CAD) system for detecting and characterizing lung nodules: Comparison with the performance of double reading by radiologists	Outcome not relate to interest
A collaborative computer aided diagnosis (C-CAD) system with eye-tracking, sparse attentional model, and deep learning	Outcome not relate to interest

End-to-end lung cancer screening with three-dimensional deep learning on low-dose chest computed tomography	Detection task
DeepLung: Deep 3D Dual Path Nets for Automated Pulmonary Nodule Detection and Classification	Cannot construct contingency table
Central focused convolutional neural networks: Developing a data-driven model for lung nodule segmentation	Outcome not relate to interest
Validation, comparison, and combination of algorithms for automatic detection of pulmonary nodules in computed tomography images: The LUNA16 challenge	Cannot construct contingency table
Automatic classification of pulmonary peri-fissural nodules in computed tomography using an ensemble of 2D views and a convolutional neural network out-of-the-box	peri-fissure nodules classification
Artificial Intelligence Assisted Computational Tomographic Detection of Lung Nodules for Prognostic Cancer Examination: A Large-Scale Clinical Trial	Detection task

Table S5. Quality assessment according to the Quality Assessment of Diagnostic Accuracy Studies 2 (QUADAS-2) criteria

	Risk of bias: QUADAS-2				Concern of applicability: QUADAS-2			Risk of bias: QUADAS-C			
	Patient selection	INDEX TEST	Reference Standard	Flow and Timing	Patient selection	INDEX TEST	Reference Standard	Patient selection	INDEX TEST	Reference Standard	Flow and Timing
Liu et al. (2023) [21]	High	Low	Low	Low	High	Low	Low	High	Low	Low	Low
Wang et al. (2022) [22]	Unclear	Low	Low	Low	Low	Low	Low	Low	Low	Low	Low
Lv et al. (2022) [23]	Unclear	Low	Low	Low	Low	Low	Low	Low	Low	Low	Low
Zhang et al. (2021) [24]	Unclear	Low	Low	Low	Low	Low	Low	Low	Low	Low	Low
Yanagawa (2021) [25]	Unclear	Low	Low	Low	Low	Low	Low	Low	Low	Low	Low
Venkadesh et al. (2021) [26]	Low	Low	Low	Low	Low	Low	Low	Low	Low	Low	Low
Sun et al. (2021) [27]	Low	Low	Low	Low	Low	Low	Low	Low	Low	Low	Low
Park et al. (2021) [28]	Unclear	Low	Low	Low	Low	Low	Low	Low	Low	Low	Low
Lv et al. (2021) [29]	Low	Low	Low	Low	Low	Low	Low	Low	Low	Low	Low
Coruh et al. (2021) [30]	Low	Low	Low	Low	Low	Low	Low	Low	Low	Low	Low
Gong et al. (2021) [31]	Unclear	Low	Low	Low	Low	Low	Low	Low	Low	Low	Low
Yang et al. (2020) [32]	Low	Low	Low	Low	Low	Low	Low	Low	Low	Low	Low
Wang et al. (2020) [33]	Unclear	Low	Low	Low	Low	Low	Low	Low	Low	Low	Low
Wan et al. (2020) [34]	Low	Low	Low	Low	Low	Low	Low	Low	Low	Low	Low
Liu et al. (2020) [35]	Low	Low	Low	Low	Low	Low	Low	Low	Low	Low	Low
Kim et al. (2020) [36]	Unclear	Low	Low	Low	Low	Low	Low	Low	Low	Low	Low
He et al. (2020) [37]	Low	Low	Low	Low	Low	Low	Low	Low	Low	Low	Low
Chae et al. (2020) [38]	Unclear	Low	Low	Low	Low	Low	Low	Low	Low	Low	Low
Zhang et al. (2019) [39]	Low	Low	Low	Low	Low	Low	Low	Low	Low	Low	Low
Wang et al. (2018) [40]	Unclear	Low	Low	Low	Low	Low	Low	Low	Low	Low	Low

Table S6. The Checklist for Artificial Intelligence in Medical Imaging scores.

Source	Title/Abstract	Introduction	Methods							Results		Discussion	Other Information	Total Score
			Study design	Data	Ground truth	Data preparation	Model	Training	Evaluation	Data	Model performance			
	(2)	(2)	(2)	(7)	(5)	(3)	(3)	(3)	(5)	(2)	(3)	(2)	(3)	(42)
Liu et al. (2023) [21]	2	2	2	4	3	2	2	1	4	1	2	2	1	28
Wang et al. (2022) [22]	2	2	2	5	4	2	2	2	4	2	1	2	1	31
Lv et al. (2022) [23]	2	2	2	5	5	2	2	2	4	2	2	2	1	33
Zhang et al. (2021) [24]	2	2	2	5	5	2	2	2	5	2	3	1	2	35
Yanagawa (2021) [25]	2	2	2	5	4	2	2	1	4	2	2	2	1	31
Venkadesh et al. (2021) [26]	2	2	2	6	4	2	2	2	5	2	2	2	2	35
Sun et al. (2021) [27]	2	2	2	5	5	2	2	2	5	2	3	2	2	36
Park et al. (2021) [28]	2	1	2	6	5	2	3	2	4	2	3	2	1	35
Lv et al. (2021) [29]	2	2	2	5	4	2	2	2	5	2	2	2	1	33
Coruh et al. (2021) [30]	2	2	2	4	4	0	2	0	3	1	1	2	1	24
Gong et al. (2021) [31]	2	2	2	5	4	2	3	3	5	1	2	2	2	35
Yang et al. (2020) [32]	2	2	2	5	4	2	1	1	3	2	1	2	1	28
Wang et al. (2020) [33]	2	2	1	4	4	2	2	2	4	1	1	1	1	27
Wan et al. (2020) [34]	2	2	2	4	3	1	1	0	3	1	2	2	1	24
Liu et al. (2020) [35]	2	2	2	4	3	1	1	0	4	2	2	2	1	26
Kim et al. (2020) [36]	2	2	2	5	5	2	2	2	5	1	2	2	1	33
He et al. (2020) [37]	2	2	1	5	4	2	1	1	3	0	1	1	1	24
Chae et al. (2020) [38]	2	2	2	4	4	2	1	1	4	1	2	2	1	28
Zhang et al. (2019) [39]	2	2	2	5	3	3	3	2	4	2	2	2	1	33

Wang et al. (2018) [40]	2	2	2	5	4	2	1	2	4	1	2	2	1	30

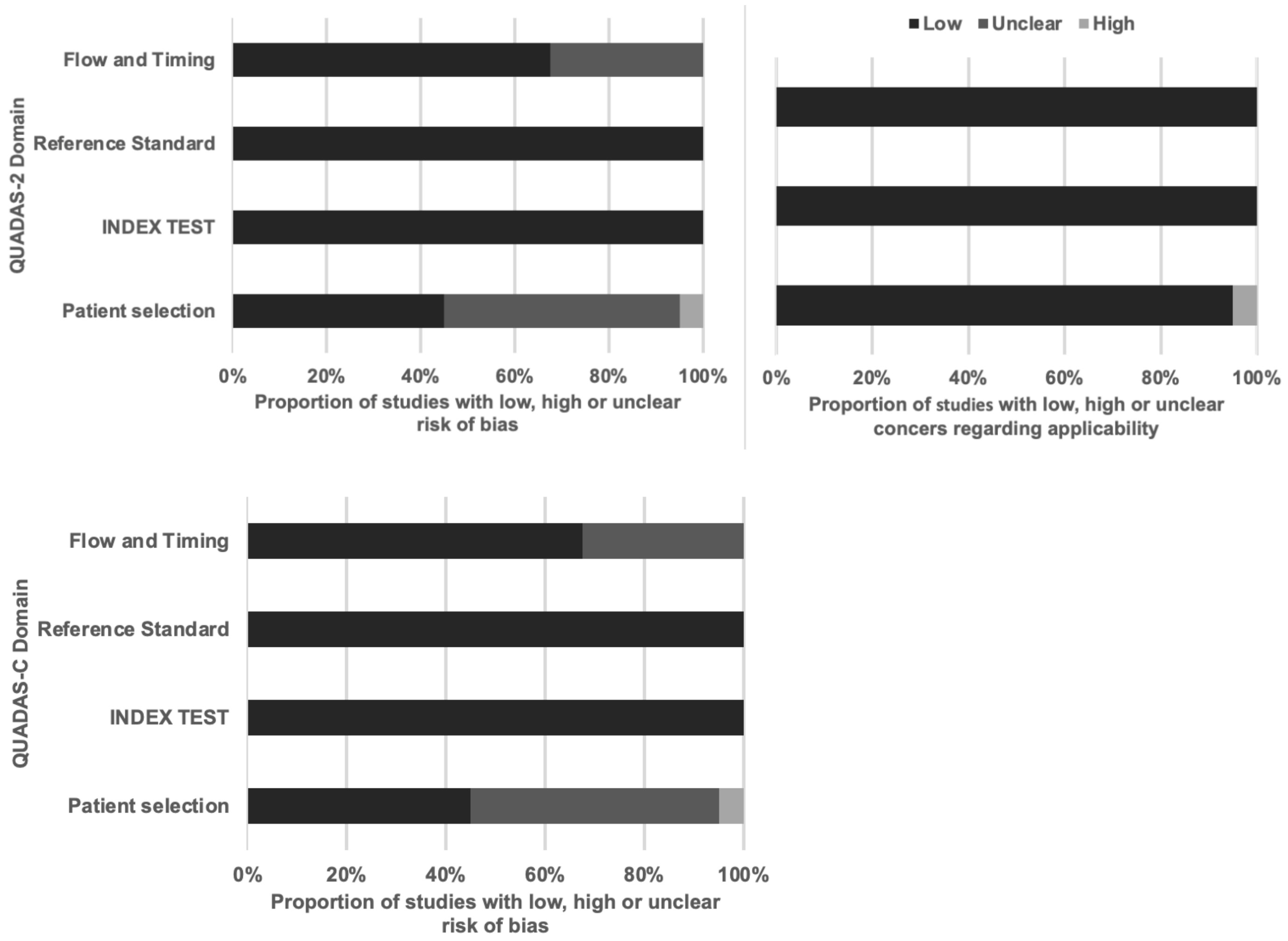

Figure S1. The results of QUADAS-2 and QUADAS-C quality assessment for included studies

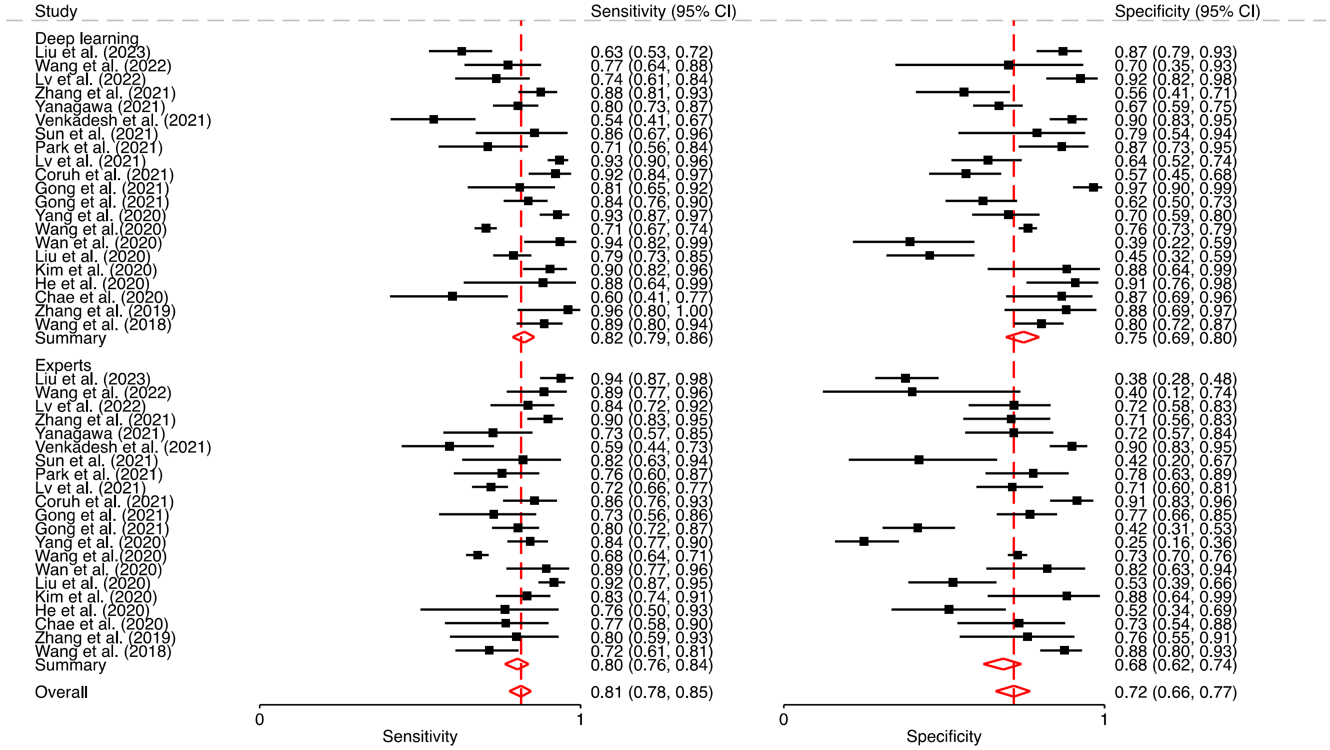

Figure S2. Forest plots show pooled estimates for chest CT

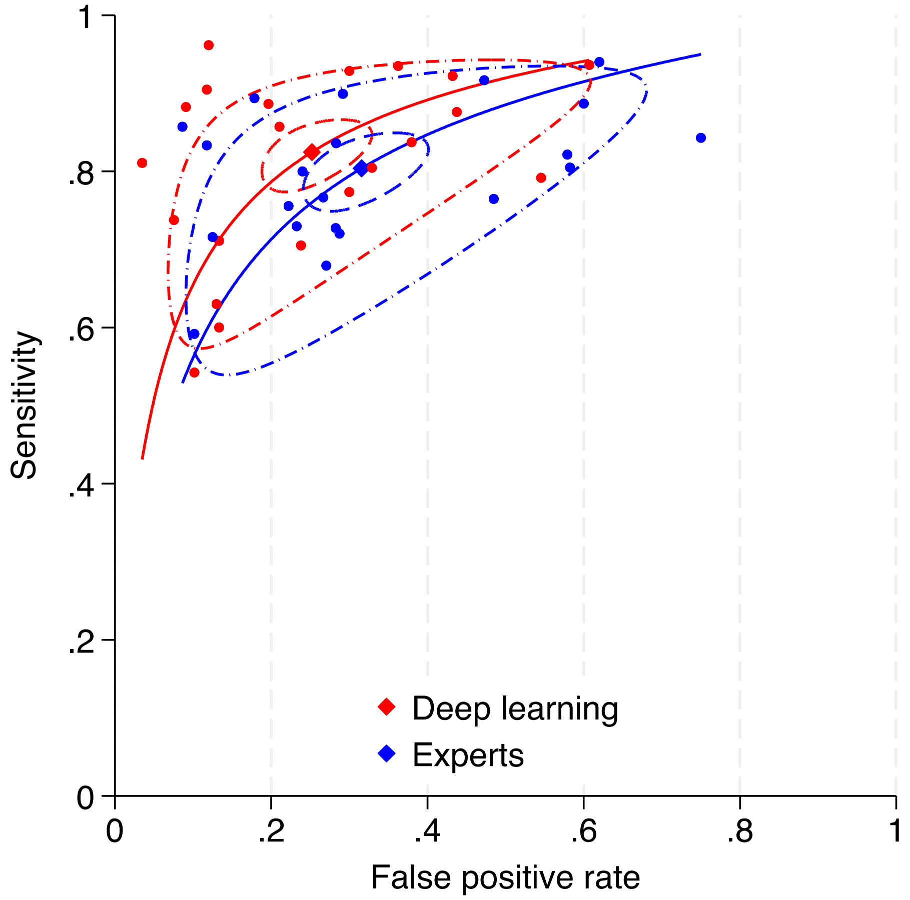

Figure S3. Summary receiver operating characteristic curves show performances of experts (blue) and deep learning (Red)

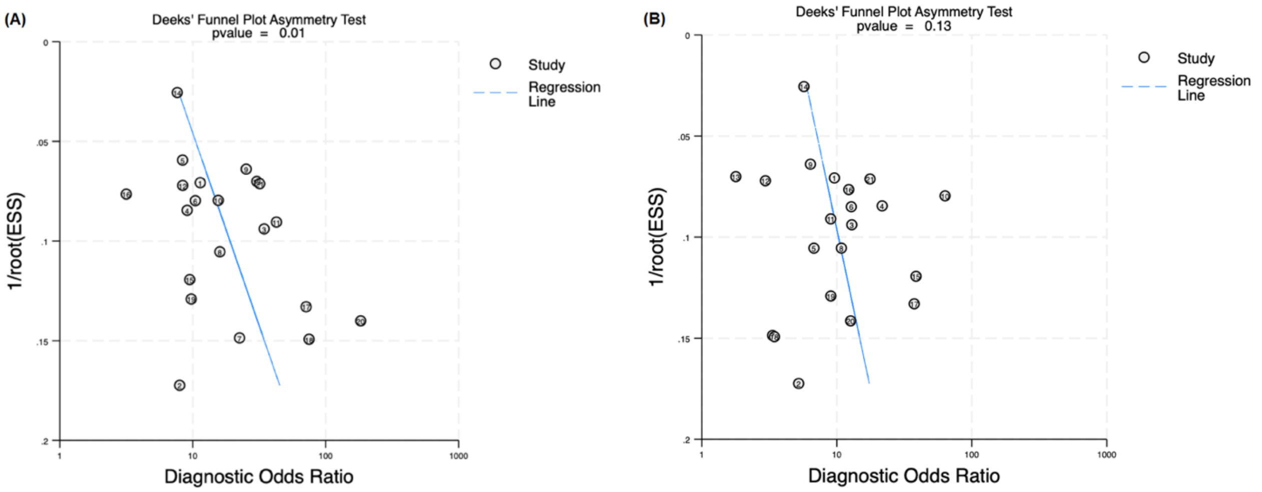

Figure S4. Deek's test plot. (A) Deep learning with CT (B) Expert with CT

*ESS: effective sample size
